# Survival in HIV-Infected Patients after a Cancer Diagnosis in the cART Era: Results of an Italian Multicenter Study

**DOI:** 10.1371/journal.pone.0094768

**Published:** 2014-04-23

**Authors:** Daria Gotti, Elena Raffetti, Laura Albini, Laura Sighinolfi, Franco Maggiolo, Elisa Di Filippo, Nicoletta Ladisa, Gioacchino Angarano, Giuseppe Lapadula, Angelo Pan, Anna Degli Esposti, Massimiliano Fabbiani, Emanuele Focà, Alfredo Scalzini, Francesco Donato, Eugenia Quiros-Roldan, the Master Cohort Group

**Affiliations:** 1 University Division of Infectious and Tropical Diseases, University of Brescia, Brescia, Italy; 2 Section of Hygiene, Epidemiology and Public Health, University of Brescia, Brescia, Italy; 3 Division of Infectious Diseases, University Hospital of Ferrara, Ferrara, Italy; 4 Division of Infectious Diseases and Unit of Antiviral Therapy, AO Papa Giovanni XXIII, Bergamo, Italy; 5 Clinic of Infectious Diseases, University of Bari, Bari, Italy; 6 Clinic of Infectious Diseases, San Gerardo de' Tintori" Hospital, Monza, Italy; 7 Clinic of Infectious Diseases, Hospital of Cremona, Cremona, Italy; 8 Clinic of Infectious Diseases, “Santa Maria Annunziata” Hospital, Firenze, Italy; 9 Institute of Clinical Infectious Diseases, Catholic University of Sacred Heart, Roma, Italy; 10 Hospital Division of Infectious and Tropical Diseases, Spedali Civili Hospital, Brescia, Italy; University of British Columbia, Canada

## Abstract

**Objectives:**

We studied survival and associated risk factors in an Italian nationwide cohort of HIV-infected individuals after an AIDS-defining cancer (ADC) or non-AIDS-defining cancer (NADC) diagnosis in the modern cART era.

**Methods:**

Multi-center, retrospective, observational study of HIV patients included in the MASTER Italian Cohort with a cancer diagnosis from January 1998 to September 2012. Malignancies were divided into ADC or NADC on the basis of the Centre for Disease Control-1993 classification. Recurrence of cancer and metastases were excluded. Survivals were estimated according to the Kaplan-Meier method and compared according to the log-rank test. Statistically significant variables at univariate analysis were entered in a multivariate Cox regression model.

**Results:**

Eight hundred and sixty-six cancer diagnoses were recorded among 13,388 subjects in the MASTER Database after 1998: 435 (51%) were ADCs and 431 (49%) were NADCs. Survival was more favorable after an ADC diagnosis than a NADC diagnosis (10-year survival: 62.7%±2.9% vs. 46%±4.2%; p = 0.017). Non-Hodgkin lymphoma had lower survival rates than patients with Kaposi sarcoma or cervical cancer (10-year survival: 48.2%±4.3% vs. 72.8%±4.0% vs. 78.5%±9.9%; p<0.001). Regarding NADCs, breast cancer showed better survival (10-year survival: 65.1%±14%) than lung cancer (1-year survival: 28%±8.7%), liver cancer (5-year survival: 31.9%±6.4%) or Hodgkin lymphoma (10-year survival: 24.8%±11.2%). Lower CD4+ count and intravenous drug use were significantly associated with decreased survival after ADCs or NADCs diagnosis. Exposure to cART was found to be associated with prolonged survival only in the case of ADCs.

**Conclusions:**

cART has improved survival in patients with an ADC diagnosis, whereas the prognosis after a diagnosis of NADCs is poor. Low CD4+ counts and intravenous drug use are risk factors for survival following a diagnosis of ADCs and Hodgkin lymphoma in the NADC group.

## Introduction

Several studies have shown that cancers are an increasingly important cause of illness and death in people with HIV [1]–[3]. Despite the link between HIV infection and cancer incidence, the question of whether HIV affects cancer prognosis in infected individuals has not been adequately addressed to date. Cancer prognosis in HIV is of interest for physicians because it can support decision-making and communication on therapeutic and palliative treatment decisions, management of co-morbid conditions, palliative care, and decisions regarding prioritization of management of other chronic conditions and/or HIV. Trends in relative incidence rates of AIDS-defining cancers (ADCs) and non-AIDS-defining cancers (NADCs) have been well characterized [4]–[9], but little is known about survival after a diagnosis of cancer in the setting of HIV infection- with the exception of non Hodgkin lymphoma (NHL), Kaposi sarcoma (KS), and anal cancer [10]–[13]. Since the introduction of combination antiretroviral therapy (cART), there has been a dramatic decrease in the incidence of AIDS-related morbidity and mortality in HIV-positive patients [14]. The cART has also improved the short- and medium-term survival in HIV-infected patients with ADCs [15]–[16] and some types of NADCs [17]. A recent Italian study has analyzed the long term survival in HIV-infected patients after malignancies [18] but few studies have investigated possible factors associated with survival in the era of cART and data in the literature are often limited to specific cancers [10]–[13]. The aim of the present work is to investigate survival after diagnosis with either ADCs or NADCs in HIV-infected patients engaged in routine care at five sites across Italy from 1998 by September 2012 and to explore possible predictors of mortality after a diagnosis of cancer in this population. The proposed study will add further information in estimates of cancers (ADCs and NADCs), their characteristics, and prognosis in the setting of HIV and cART, to better understand the challenges that this booming population poses to oncologic and infectious health services in the near future.

## Materials and Methods

### MASTER cohort and study population

The MASTER (MAnagement Standardizzato di TERapia antiretrovirale) cohort started in 1997 in several HIV outpatient clinics across Italy (Brescia, Bergamo, Monza, Cremona, Ferrara, Firenze, Roma, Bari), with the following aims (i) to create a population-based nationwide database of HIV patients for scientific projects, (ii) to monitor the spread and demographic characteristics of the HIV epidemic in Italy, (iii) to monitor and compare the effects of antiretroviral strategies at a national level. Patients medical records were retrospectively traced back to 1986 and prospectively followed up until now. (http://www.mastercohort.it). Enrollment in MASTER is independent of the stage of disease, the degree of immune suppression, or whether the individual is receiving antiretroviral therapy. Comorbid conditions (*i.e.* diabetes mellitus, hypertension, cardiovascular disease, cancer diagnosis, substance abuse, liver and renal conditions) and causes of death are accurately reported. Laboratory data, including CD4 T-cell count and HIV viral load, are collected at each patient's visit. Data are recorded over a standardized time-scale every three/four months in a common electronic chart (NetCare or Health&Notes). Data merging and cleaning are performed at a central level every six months.

Here, we conducted a retrospective cohort study from January 1998 to December 2012 on HIV-infected patients with a diagnosis of cancer, either naïve or experienced to antiretroviral therapy, with the following available records: date of first HIV positive test or cohort-entry, date of death (for patients who died during the study period), date of last visit (for patients still alive or lost to follow-up), and at least one record of CD4+ T-cell count available. A follow-up period of at least 1 day was required. Patients without any data recorded during 1 year or longer have been considered lost to follow-up. Measures for CD4+ T-cell count performed within 3 months before or after of the date of cancer diagnosis were reported as referred to the time of cancer diagnosis.

### Ethics Statement

At first visit, patients provide written informed consent to include their clinical and biological data in the MASTER database for scientific purpose. The data was anonymized before it was provided and the database is hosted in Fondazione MISI's headquarter in compliance with current regulations.

The study was approved by the Ethical Committee of the Hospital Spedali Civili, Brescia (Coordinating Centre) and those of the following Institutions: University Hospital of Ferrara, Ferrara; AO Papa Giovanni XXIII, Bergamo; University of Bari, Bari; San Gerardo de' Tintori" Hospital, Monza; Hospital of Cremona, Cremona; “Santa Maria Annunziata” Hospital, Firenze; University of Sacred Heart, Roma.

### Cancer diagnoses and deaths

Malignant cancer diagnoses were collected from medical records and verified through a standardized process, including detailed record abstraction and adjudication of malignancies. Only incident cancer events that occurred during the follow-up were included in the analysis. Recurrence of cancer and metastases were excluded. Cancer type or cancer site were coded according to the WHO classification [19] and malignancies were defined as ADCs (Non-Hodgkin lymphoma, Kaposi sarcoma and invasive cervical carcinoma) and NADCs (all other types of cancers). Primary central nervous system lymphoma (PCNSL) and systemic non-Hodgkin lymphomas were grouped together. Death and date of death were ascertained by centers through chart review and in some, cross-checks with mortality registers.

### Statistical analysis

Values are reported as medians (interquartile range, IQR) or frequencies (%), as appropriate. Quantitative variables were compared by the non-parametric Mann-Whitney rank-sum test; the χ^2^test was used to assess independence between qualitative variables.

For each patient included in the study, person-years at risk have been calculated starting from the date of cancer diagnosis. The observation period ended on December 31st, 2012, or last follow-up visit, or death, whichever occurred first. According to cancer occurrence patients were classified in 2 groups: i) patients who developed ADC; ii) patients who developed NADC. Multiple primaries, i.e. cancers of a different type occurring in the same subject, were included in the analysis and each cancer was considered as a single case. Cancer incidence rates (IRs) were computed for ADC and NADC, dividing observed cases by the corresponding person-years at risk, and standardized for sex and age using the direct method with the European population as the standard and truncated at 65 years-old. The rates were expressed per 1,000 person-years. To compare the incidence of specific cancers in our HIV-infected patients with that observed in the Italian general population, the standardized incidence ratios (SIRs) and their corresponding 95% confidence intervals (with the Byar's approximation of Poisson model) were calculated using the number of expected cases based on the general population gender- and age-specific rates for Italy provided by 5 Italian Cancer Provincial Registries (Torino, Varese, Ferrara, Latina, Ragusa). SIRs were calculated until 2007, since these registries are updated until this year.

Survival was determined from the date of diagnosis of cancer to the end of the follow up, corresponding to the end of observation period. The probabilities of survival were estimated at 1, 5 and 10 years according to Kaplan-Meier with Greenwood standard error (SE) for total ADCs, total NADCs and specific cancers. Relative survival and expected survival were estimated according to the Ederer II method from life-tables for all-cause mortality by age, gender and calendar year. In the case of patients who had both ADC and NADC diagnosis, survival analysis was performed on the first cancer diagnosed. The factors associated with all-cause mortality were identified using the log–rank test for univariate analyses. Furthermore, the same variables were tested by multivariate analysis using Cox proporzional harzards models. First, fully adjusted models were fitted including the following variables: age, gender, cART, year of cancer diagnosis (categorized as 1998–2002, 2003–2007, 2008–2012), HIV viral loads and CD4 counts at cancer diagnosis, previous AIDS event, and risk factor for HIV acquisition. Afterwards, a selection of variables was performed with a stepwise backward procedure to generate parsimonious models. Age and gender were included as possible confounders, regardless of statistical significance. Results are shown with estimated hazards ratios (HR), 95% CIs, and P values (according to the Wald test). The proportional hazard assumption was assessed for each variable either graphically (by examining the log-log survival plot and the comparison of “observed” with “expected” survival curves) or by the goodness of fit approach. All the statistical tests were two-sided, assumed a level of significance of 0.05 and were performed using the STATA 12 software (STATA Statistics/Data Analysis 12.0 - STATA Corporation, College Station, TX, USA).

## Results

### Characteristics of Master Cohort and cancer incidence

From January 1998, a total of 13,388 patients have been included in the Master database and followed during 96,228 person-years (PY). Over this time a total of 900 cancer diagnosis were recorded: 454 ADCs (Incidence rate [IR]: 4.2/1000 PY, 95% confidence interval [CI] [3.7–4.8]) and 446 NADCs (IR: 4.6/1000 PY, 95%CI 3.9–5.3). A total of 27 patients had multiple cancer diagnosis. IRs were also calculated after stratification by calendar period (1998–2002; 2003–2007; 2008–2012). As expected, in our cohort the incidence rates in ADC had decreased in the last years (IR 1998–2002: 6.6/1000 PY, 95%CI 4.7–8.4; IR 2003–2007:5.3/1000 PY, 95%CI 4.0–6.7; IR 2008–2012: 3.4/1000 PY, 95%CI 2.5–4.2) whereas the incidence rates in NADC remained almost stable over time (IR 1998–2002: 5.7/1000 PY, 95%CI 3.0–8.5; IR 2003–2007:4.4/1000 PY, 95%CI3.6–5.3; IR 2008–2012: 4.4/1000 PY, 95%CI 3.4–5.4). Although the global incidence for NADCs resulted comparable to that detected in the Italian general population (SIR 1.1 [95%CI 1.0–1.2]), we observed higher SIRs for liver cancer (SIR 15.1 [95%CI 11.4–19.6]), Hodgkin lymphoma (SIR 14.2 [95%CI 10.1–19.4]), and a two-fold higher incidence also for lung cancer (SIR 2.2 [95%CI 1.5–3.2]). Breast cancer incidence was similar to the general population (SIR 1.3 [95%CI 0.8–1.9]).

### Survival following ADC and NADC diagnosis

For the survival analysis we excluded 34 patients with a post-mortem cancer diagnosis or with a follow-up after a cancer diagnosis <1 day. Therefore 866 patients with malignancies were included in the analysis: 435 (51%) patients with an ADC and 431 (49%) with a NADC diagnosis. Table 1 summarizes the patient's characteristics at cancer diagnosis.

**Table 1:** Patients' characteristics at cancer diagnosis.

Variable	Categories	ADCs (n = 435)	NADCs (n = 431)	Overall (n = 866)	P-value
**Death, n (%)**		142 (33)	166 (38)	308 (36)	0.071
**Gender, n (%)**					0.372
	Male	345 (79)	331 (77)	676 (78)	
	Female	90 (21)	100 (23)	190 (22)	
**Age at cancer diagnosis, years, n (%)**					<0.001
	18–34	82 (19)	36 (8)	118 (14)	
	35–49	258 (59)	251 (58)	509 (59)	
	≥50	95 (22)	144 (33)	239 (27)	
**HIV risk factor, n (%)**					0.001
	IVDU	140 (32)	185 (43)	325 (38)	
	other	295 (68)	246 (57)	541 (62)	
**HCV or HBV co-infection, n (%)**		183 (42)	238 (55)	421 (49)	<0.001
**Previous AIDS event, n (%)**		184 (42)	183 (42)	367 (42)	0.962
**CD4 cell count at cancer diagnosis, cell/mm^3^**					<0.001
	≥200	203 (46)	310 (72)	513 (59)	
	<200	181 (42)	103 (24)	284 (33)	
	missing	51 (12)	18 (4)	69 (8)	
**Nadir Cd4, cell/mm^3^, n(%)**					<0.001
	≥200	113 (26)	176 (41)	289 (33)	
	100–199	187 (43)	170 (39)	357 (41)	
	<50	135 (31)	85 (20)	220 (26)	
**Antiretroviral therapy, n (%)**					0.007
	No cART	80 (18)	51 (12)	131 (15)	
	cART	355 (82)	380 (88)	735 (85)	
**HIVRNA, copies/mL, n (%)**					<0.001
	Undetectable	107 (25)	198 (46)	305 (36)	
	Positive	267 (61)	213 (49)	480 (55)	
	missing	61 (14)	20 (5)	81 (9)	
**Median age at diagnosis (IQR)**		42 (36–48)	48 (41–54)	44 (38–50)	<0.001
**Median CD4 cell count (IQR)**		214(85–409)	349 (200–498)	291 (133–457)	<0.001
**Median HIVRNA, copies/mL**		657 (37–21000)	70 (37–4300)	301 (37–9400)	<0.001

Note: ADCs: AIDS-defining cancers, NADCs: Non-AIDS-defining cancers, IVDU: injection drug users, HBV: hepatitis B virus, HCV: hepatitis C virus, cART: combined antiretroviral therapy.

Among ADCs, 184 (42%) patients were diagnosed with Kaposi sarcoma, 34 (8%) patients with cervical cancer and 217 (50%) patients with Non-Hodgkin lymphoma (NHL). Most of the patients (77%) diagnosed with cervical cancer had a CD4+ T-cell count >200 cells/mm^3^ and almost half of them (44%) had a nadir CD4+ count >200 cell/mm^3^. At the time of analysis, patients with non-Hodgkin lymphoma had a higher number of deaths (n = 97, 45%) compared to those with Kaposi sarcoma (n = 40, 22%) and invasive cervical carcinoma (n = 5, 15%, p<0.001).

Liver cancer was the most frequent NADCs (n = 69, 16%), followed by Hodgkin lymphoma (n = 61, 14%), lung cancer (n = 35, 8%) and breast cancer (n = 30, 7%). Patients with Hodgkin lymphoma and breast cancer tended to be younger at diagnosis than those with other cancer (median age: 42 years vs. 47 and 52 years for liver and lung cancer, respectively). At the time of NADC diagnosis, 75% of the patients had a CD4+ T-cell count >200 cells/mm^3^. Fewer number of deaths were observed at the time of analysis among the patients diagnosed with breast cancer (n = 6, 20%) compared to the patients with Hodgkin lymphoma (n = 26, 43%) and those with liver or lung cancers (n = 44, 64% and n = 22, 63%, respectively, p<0.001).

**Figure 1:**
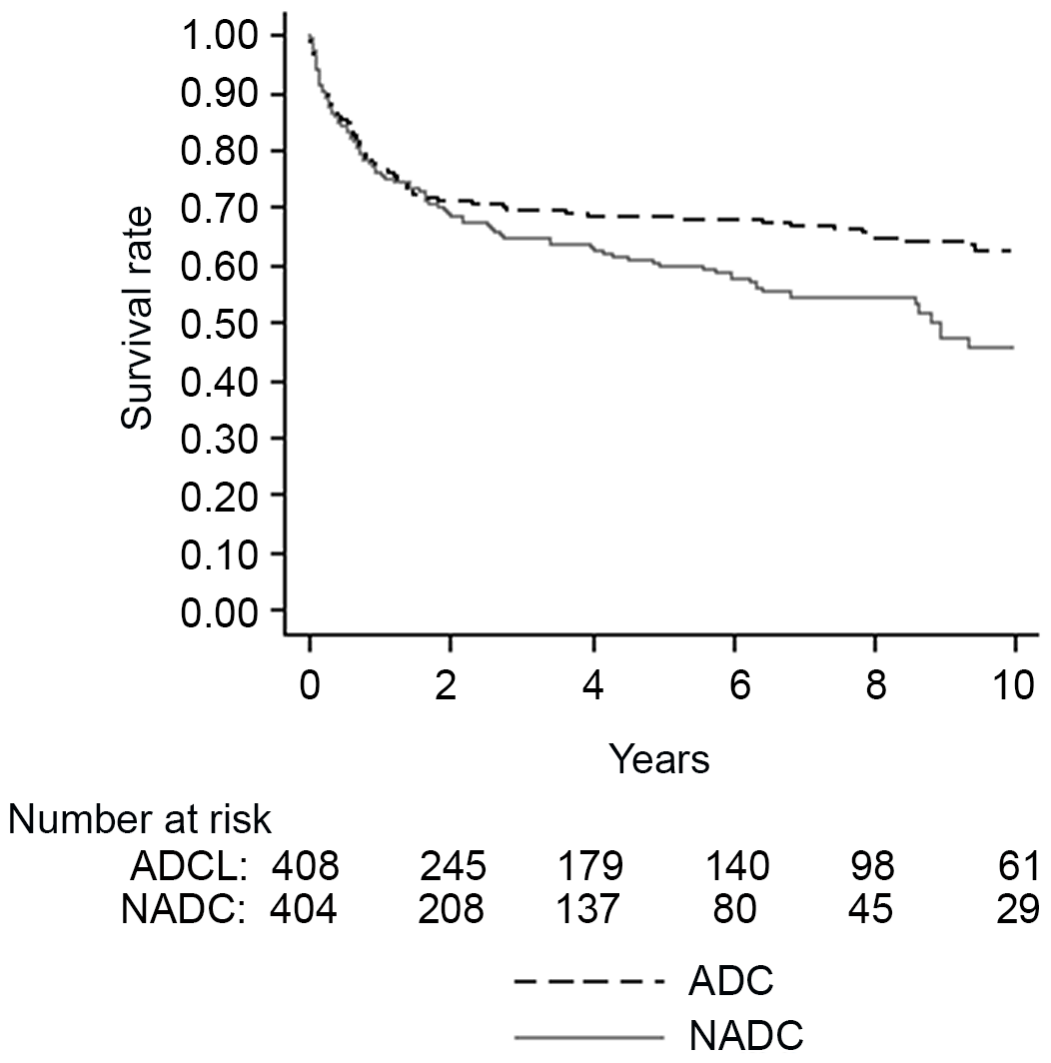
Survival probabilities according to cancer classification. ADC, AIDS-defining cancer. NADC, non-AIDS-defining cancer.

Figure 1 shows the 10-years survival analysis after an ADC or NADC diagnosis. Although in the first 2 years after the cancer diagnosis no statistically significant difference in survival between ADC and NADC was found (p = 0.474), patients with an ADC diagnosis had a significantly higher long-term survival probability compared to those with a NADC diagnosis (10-year survival: 62.7%±2.9% vs. 46%±4.2%; p = 0.017). One year survival was also analyzed in relation to the time of cancer diagnosis. HIV-infected patients with a more recent NADC diagnosis had a statistically significant higher survival rate: one-year survival in patients with NADC diagnosis after 2008 was 80% vs. 76% and 65% in patients with NADC diagnosis between the period 2002–2007 and before 2002, respectively (P = 0.047).

**Figure 2:**
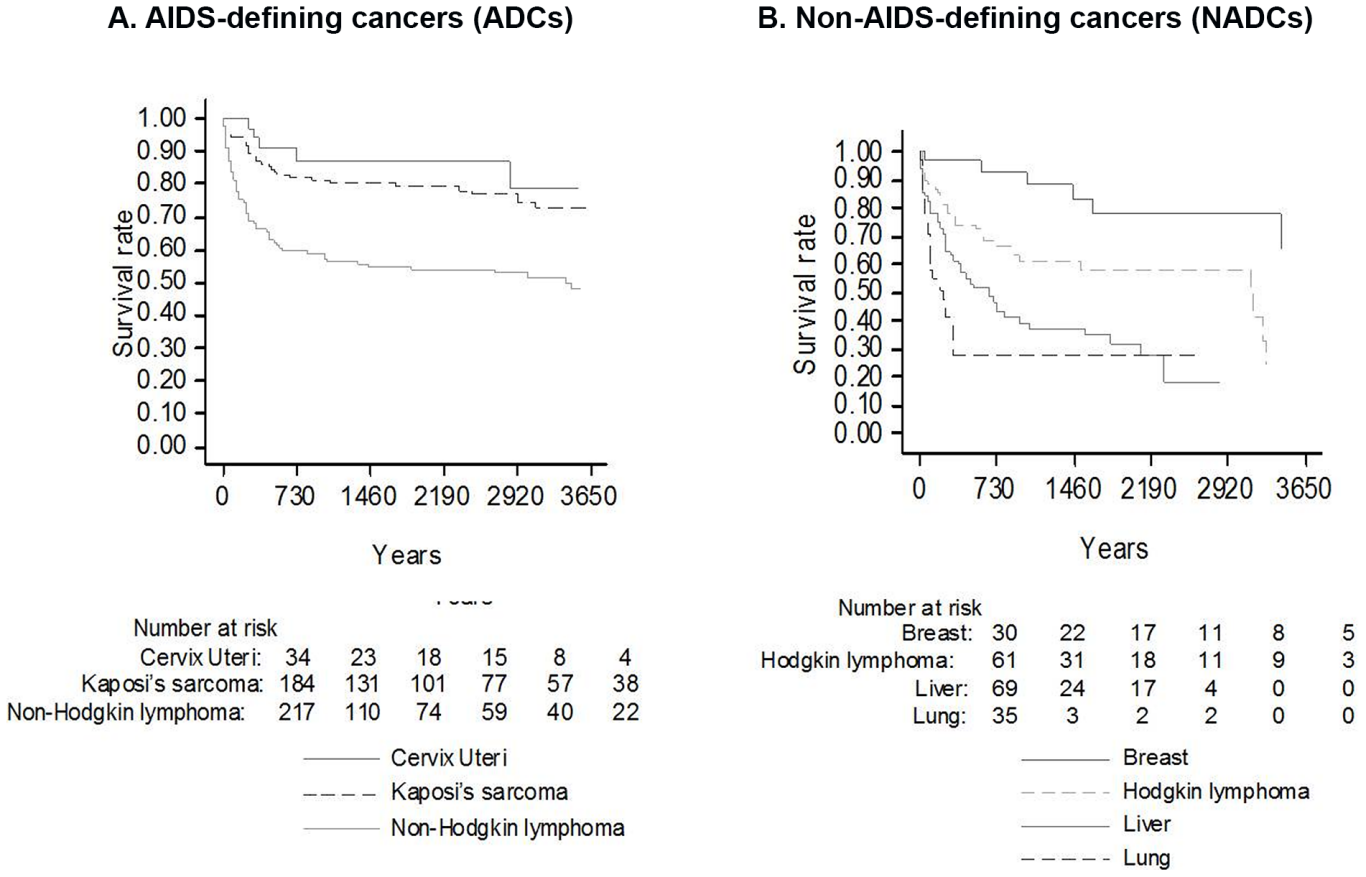
Survival curves according to specific cancer types. Panel (A) Survival probabilities according to AIDS-defining cancer (ADC) diagnosis. Overall, the median survival time from diagnosis of all ADC was 3.4 years; more specifically, the median survival time of NHL was 2.1 years, Kaposi sarcoma 4.7 years, and cervical cancer 5.1 years. Panel (B) Survival probabilities according to non-AIDS-defining cancer (NADC) diagnosis. The overall median survival time of NADC was 1.6 years; more specifically, the median survival time of liver cancer was 441 days, lung cancer 113 days, breast cancer 1624 days, and Hodgkin lymphoma 795 days.

Survival in patients diagnosed with the different types of ADCs and with the most representative NADCs are showed in Figure 2 (Panel A and Panel B).

### Predictors of mortality

Risk factors for poorer survival after ADCs in univariate analysis were, as expected, viro-immunological variables (lower nadir- and CD4+ T-cell count at diagnosis, detectable HIVRNA, previous AIDS event) and IDVU mode of infection (Table 2). Notably, after a cervical cancer diagnosis only lower CD4+ T-cell count was associated with decreased survival rates (Table 2).

**Table 2:** Rate of overall survival ADCs by patients' characteristics.

Variables	Categories	ADCs (n = 435)			P-value	CERVICAL CA (n = 34)			P-value	KAPOSI SARCOMA (n = 184)			P-value	NON HODGKIN LYMPHOMA (n = 217)			P-value
		1y	5y	10y		1y	5y	10y		1y	5y	10y		1y	5y	10y	
**Total**		77.0±2.0	67.7±2.4	61.1±2.9		90.9±5.0	87.3±6.0	78.5±9.9		86.9±2.5	79.1±3.2	72.8±4.0		66.6±3.2	55.0±3.5	48.2±4.3	
**Gender**					0.613				NE*				0.498				0.874
	Male	77.8±2.3	66.7±2.7	60.5±3.2		-	-	-		87.7±2.6	79.2±3.3	73.5±4.1		66.9±3.6	54.6±4.0	47.4±4.9	
	Female	76.8±4.5	71.7±4.9	63.8±6.1		90.9±5.0	87.3±6.0	78.5±9.9		76.9±11.7	76.9±11.7	65.9±14.3		65.1±7.6	57.0±8.0	51.3±9.0	
**Age, years**					0.372				0.751				0.911				0.619
	18–34	82.4±4.3	76.2±5.0	65.1±6.7		100	100	66.7±2.7		89.6±4.9	83.7±6.1	69.6±9.0		67.1±8.5	57.8±9.7	57.8±9.7	
	35–49	74.9±2.8	65.7±3.1	59.8±3.7		85.7±7.6	80.4±8.8	80.4±8.8		84.7±3.8	76.0±4.6	73.6±5.0		66.4±4.0	56.1±4.4	46.4±5.5	
	50-max	77.8±4.3	65.6±5.1	61.5±6.2		100	-	-		89.0±4.6	81.5±6.0	74.1±8.9		66.7±6.8	49.7±7.6	49.7±7.6	
**IDVU**					<0.001				0.060				0.002				0.276
	No	80.7±2.3	73.2±2.7	67.7±3.5		91.3±5.9	91.3±5.9	73.0±17.0		90.0±2.5	83.4±3.3	77.8±4.4		68.0±4.2	58.1±4.7	54.6±5.5	
	Yes	69.3±3.9	56.4±4.3	48.6±4.8		90.0±9.5	80.0±12.6	80.0±12.6		74.8±7.3	62.5±8.3	53.8±9.1		64.7±5.0	51.4±5.4	42.0±6.2	
**AIDS event**					0.024				0.273				0.162				0.022
	No	79.0±2.6	73.4±2.9	65.4±4.0		90.5±6.4	90.5±6.4	90.5±6.4		88.8±3.3	86.3±3.7	74.8±6.3		70.5±4.0	61.8±4.3	55.5±5.8	
	Yes	74.3±3.3	60.4±3.8	55.2±4.1		91.7±8.0	81.5±11.9	61.1±19.8		85.0±3.8	72.2±5.0	70.2±5.2		60.2±5.5	44.4±5.8	37.8±6.1	
**Coinfection**					0.075				0.957				0.129				0.895
	No	78.6±2.6	70.8±3.0	67.4±3.5		85.7±9.3	85.7±9.3	85.7±9.3		88.8±2.8	82.1±3.6	76.5±4.6		65.5±4.7	55.5±5.0	55.5±5.0	
	Yes	75.0±3.2	63.61±3.7	53.7±4.5		94.7±5.1	88.8±7.5	76.1±13.4		82.8±5.2	72.5±6.3	64.7±7.7		67.7±4.5	54.8±5.0	44.2±6.0	
**cART**					0.001				0.660				0.007				0.024
	No	63.7±5.5	51.7±5.9	49.6±6.0		90.0±9.5	90.0±9.5	90.0±9.5		67.4±9.5	58.9±10.0	58.9±10.0		55.5±7.7	39.2±7.8	35.9±7.8	
	Yes	80.0±2.2	71.3±2.5	63.6±3.2		91.3±5.9	86.5±7.3	74.1±13.0		90.0±2.4	82.4±3.2	75.0±4.3		69.4±3.5	59.3±3.9	51.7±4.9	
**CD4+ count, cell/mm^3^**					<0.001				0.003				0.001				0.001
	200-max	84.3±2.6	76.6±3.2	70.6±4.2		96.1±3.8	96.1±3.8	96.1±3.8		94.1±2.9	89.6±4.1	86.2±5.2		74.6±4.3	62.9±5.0	53.2±6.9	
	0–199	64.3±3.6	53.7±3.9	49.3±4.1		71.4±17.1	57.1±18.7	42.9±18.7		78.8±4.6	69.1±5.3	64.3±5.9		50.8±5.4	39.5±5.3	36.9±5.6	
**Nadir CD4+ count, cell/mm^3^**					<0.001				0.085				0.001				0.033
	≥200	87.4±3.2	81.3±3.8	77.9±4.9		100	100	100		97.9±2.1	93.0±3.9	85.9±7.8		73.3±6.3	63.9±7.1	63.9±7.1	
	50–199	69.3±3.5	77.7±3.1	61.4±4.4		75.0±15.3	62.5±17.1	62.5±17.1		88.8±3.7	84.0±4.5	75.6±6.1		69.8±4.6	59.2±5.1	49.9±6.6	
	<50	67.5±4.1	54.5±4.5	48.8±4.9		90.0±9.5	90.0±9.5	90.0±9.5		75.5±5.7	61.9±6.6	58.8±7.0		56.8±6.2	42.8±6.3	37.1±6.8	
**HIVRNA, copies/mL**					0.020				0.841				0.015				0.032
	Undetectable	84.8±3.5	77.8±4.3	72.6±5.3		91.7±7.9	91.7±7.9	61.1±25.5		97.6±2.3	91.2±5.0	86.1±6.8		73.2±6.1	64.4±6.8	64.4±6.8	
	Positive	70.8±2.8	60.0±3.2	54.8±3.6		90.5±6.4	84.8±8.1	84.8±8.1		80.4±3.9	71.7±4.6	67.5±5.2		60.2±4.2	47.1±4.5	40.2±5.1	

Note: Plus minus are value standard error, P value are from long rank test. - The sample size was too small for Kaplan Meier analysis.

*NE, not evaluable. ADCs: AIDS-defining cancers, IVDU: Intravenous Drug Use, cART: combined antiretroviral therapy; CA: cancer.

In univariate analyses, poorer survival after NADC diagnosis was associated with intravenous drug use (IVDU) mode of infection, lower CD4+ T-cell count at NADC diagnosis and previous AIDS event (Table 3). Analyses of risk factors for mortality after diagnosis of specific NADC were limited due to the relatively small number of patients experiencing these events. However, male gender, age between 35–49, previous AIDS event, lower CD4+cell count at diagnosis and lower nadir CD4+ T-cell count were associated with mortality after Hodgkin lymphoma. No association was observed between type of NADCs and epidemiologic and clinical variable among the patients with lung, liver or breast cancers (Table 3).

**Table 3:** Rate of overall survival NADCs by patients' characteristics.

Variables	Categories	NADCs (n = 431)			P-value	LIVER CA (n = 69)			P-value	LUNG CA (n = 35)		P-value	BREAST CA (n = 30)			P-value	HODGKIN LYMPHOMA (n = 61)			P-value
		1y	5y	10y		1y	5y	10y		6month	1y		3y	5y	10y		1y	5y	10y	
**Total**		76.1±2.1	59.2±2.7	45.0±4.1		59.8±6.0	31.9±6.4	-		54.4±8.7	28.0±8.6		96.7±3.3	78.1±8.9	65.1±14.0		74.2±5.7	57.8±7.2	24.8±11.2	
**Gender**					0.152				0.380			NE*				0.089				0.003
	Male	74.7±2.4	56.8±3.1	40.9±4.8		56.0±6.5	27.4±6.8	-		58.3±9.3	32.0±9.6		80.0±17.9	53.3±24.8	26.7±22.6		77.0±5.8	63.0±7.5	27.1±12.2	
	Female	80.4±4.0	67.4±5.1	58.6±6.5		87.5±11.7	62.5±17.1	-		-	-		90.3±6.6	83.9±8.7	83.9±8.7		50.0±20.4	-	-	
**Age, years**					0.331				0.096			0.094				0.108				0.045
	18–34	82.3±6.6	68.4±8.4	62.1±9.6		-	-			-	-		66.7±2.7	33.3±2.7	33.3±2.7		83.1±11.0	71.2±14.5	71.2±14.5	
	35–49	76.2±2.7	58.1±3.5	42.4±5.3		67.9±6.8	33.2±7.5	-		42.8±13.2	14.3±9.3		88.2±7.9	80.9±10.1	53.9±23.0		68.3±7.6	49.5±8.6	9.9±9.0	
	50-max	74.2±3.8	59.4±5.0	44.5±8.1		40.9±11.1	35.1±10.9	-		81.6±5.3	71.1±6.4		-	-	-		88.9±10.5	88.9±10.5	88.9±10.5	
**IVDU**					0.007				0.753			0.606				0.292				0.686
	No	81.7±2.5	63.1±3.7	53.5±5.4		60.5±13.8	32.3±16.7	-		57.9±12.2	30.4±13.4		86.5±7.3	80.8±8.8	80.8±8.8		78.1±7.3	60.2±9.8	60.2±9.8	
	Yes	68.7±3.5	54.1±4.0	37.2±5.5		59.6±6.7	31.8±6.7	-		50.9±12.5	25.5±11.0		100	66.7±2.7	-		70.0±8.9	56.1±10.2	18.7±11.3	
**AIDS event**					0.008				0.683			0.074				0.480				0.021
	No	78.5±2.7	67.4±3.3	48.6±6.0		56.8±7.9	40.5±8.4	-		56.7±10.9	39.2±11.4		92.9±6.8	85.1±9.7	63.8±19.8		82.2±7.3	74.8±9.7	24.9±20.6	
	Yes	72.8±3.4	48.6±4.3	39.7±5.4		64.3±9.1	22.7±9.3	-		50.3±14.4	-		82.5±11.3	68.7±15.7	68.7±15.7		65.5±8.8	41.3±9.7	20.7±11.4	
**Coinfection**					0.146				NE*			0.295				0.850				0.699
	No	78.1±3.1	62.2±4.1	52.1±6.1		-	-			63.6±11.1	33.9±12.6		83.5±10.8	74.3±13.0	74.3±13.0		64.5±9.1	59.6±9.7	59.6±9.7	
	Yes	74.5±2.9	56.9±3.6	40.6±5.2		59.8±6.0	31.9±6.4			42.9±13.2	21.4±11.0		92.9±6.9	79.6±13.6	39.8±28.9		83.3±6.8	59.0±9.9	19.7±11.8	
**cART**					0.152				0.572			NE*				NE*				0.532
	No	71.9±6.4	47.2±7.4	43.9±7.6		57.1±16.4	45.7±16.6	-		-	-		-	-	-		80.0±17.9	40.0±21.9	-	
	Yes	76.7±2.2	61.0±2.9	45.1±4.5		60.2±6.5	29.0±7.0	-		57.0±9.4	34.1±9.9		96.7±3.3	78.1±8.9	65.1±14.0		73.7±6.1	60.4±7.5	25.9±11.7	
**CD4+ T-cell count, cell/mm^3^**					0.014				0.225			NE*				0.270				0.006
	200-max	78.1±2.4	62.4±3.2	48.7±5.1		61.6±6.9	38.8±8.2	-		61.7±9.6	32.1±10.4		91.7±8.0	81.5±11.9	81.5±11.9		83.2±5.8	63.3±8.6	25.3±14.3	
	0–199	68.7±4.7	48.8±5.3	32.7±7.0		59.2±11.9	17.8±9.3	-		-	-		80.0±12.6	68.6±15.1	51.4±18.7		50.0±13.4	40.0±13.4	-	
**Nadir CD4+ counts, cell/mm^3^**					0.337				0.891			0.427				0.433				0.083
	≥200	76.2±3.3	62.9±4.3	51.6±6.8		53.1±9.9	31.9±12.1	-		64.0±10.9	33.9±11.5		90.9±8.7	90.9±8.7	90.9±8.7		81.9±9.5	51.6±14.0	-	
	50–199	78.4±3.2	57.9±4.3	42.9±6.0		61.0±8.5	30.5±8.3	-		57.1±16.4	21.4±17.5		85.7±13.2	64.3±2.1	64.3±2.1		78.6±7.7	50.6±8.4	40.7±16.0	
	<50	71.1±5.0	54.4±6.2	39.5±8.8		75.0±15.3	37.5±17.1	-		-	-		87.5±11.7	72.9±16.5	48.6±22.7		52.7±14.1	44.0±14.3	-	
**HIVRNA, copies/mL**					0.110				0.149			NE*				0.668				0.233
	Undetectable	79.8±2.9	60.0±4.6	48.5±7.6		69.9±7.6	35.9±9.9	-		72.3±10.6	47.9±13.7		100	53.3±24.8	-		85.0±7.9	67.7±13.2	-	
	Positive	71.7±3.2	55.8±3.7	41.7±4.9		49.0±9.3	26.4±8.4	-		-	-		81.7±9.6	81.7±9.6	81.7±9.6		68.7±7.9	55.1±8.8	22.0±12.6	

Note: Plus minus are value standard error, P value are from long rank test. - The sample size was too small for Kaplan Meier analysis.

*NE, not evaluable.

NADCs: Non-AIDS-defining cancers, IVDU: Intravenous Drug Use, cART: combined antiretroviral therapy; CA: cancer.

In multivariable analyses, predictors of lower survival probability after both ADCs and NADCs diagnosis were lower CD4+ T-cell count and IVDU as mode of HIV infection (Table 4). Being on cART at cancer diagnosis was associated with improved survival after ADCs. A previous AIDS event was no longer associated with survival after both ADCs and NADCs when imputed with the other covariates in the multivariable model. For ADC, the association between the HIV RNA serum levels at the time of cancer diagnosis and mortality risk was next to threshold for statistical significance (HR: 1.6, 95%CI 0.99–2.53, p = 0.060). Multivariate analyses of risk factors for mortality after diagnosis of specific ADC or NADC cancers are shown in Table 5.

**Table 4:** Cox regression multivariate models for ADCs and NADCs.

Variable	Categories	ADCs		NADCs	
		HR (95%CI)	P-value	HR (95%CI)	P-value
**Gender**	Male vs Female	1.4 (0.86–2.20)	0.179	1.2 (0.78–1.78)	0.443
**Age, years**					
	18–34Rif	1Rif		1Rif	-
	35–49	1.3 (0.77–2.15)	0.338	1.2 (0.62–2.39)	0.576
	50-max	1.7 (0.94–3.19)	0.077	1.8 (0.91–3.68)	0.092
**Years of Diagnosis**					
	1998–2002	1Rif		1Rif	-
	2003–2007	1.15 (0.77–1.71)	0.487	1.2 (0.81–1.82)	0.354
	2008–2012	0.88 (0.51–1.51)	0.641	0.8 (0.49–1.32)	0.398
**cART Therapy**	Yes vs No	0.5 (0.34–0.82)	0.004	0.8 (0.52–1.33)	0.452
**CD4+ count at diagnosis, cell/mm^3^**	0–199 vs 200-max	2.4 (1.59–3.49)	<0.001	1.5 (1.05–2.08)	0.025
**IVDU**	Yes vs No	1.9 (1.30–2.77)	0.001	1.6 (1.13–2.34)	0.008
**HIV RNA at diagnosis**	Positive vs. undetectable	1.6 (0.99–2.53)	0.060	1.2 (0.85–1.74)	0.281
**Previous AIDS event**	Yes vs No	1.0 (0.70–1.46)	0.944	1.2 (0.91–1.74)	0.168

Note: ADCs: AIDS-defining cancers, NADCs: Non-AIDS-defining cancers, IVDU: injection drug users, cART: combined antiretroviral therapy; HR: Hazard ratio; CI: confidence interval.

**Table 5:** Cox regression multivariate models for ADC and NADC specific cancers.

Variable	Categories	ADCs						NADCs							
		CERVICAL CA		KS		NHL		LIVER CA		LUNG CA		BREAST CA		HL	
		HR (95%CI)	P-value	HR (95%CI)	P-value	HR (95%CI)	P-value	HR 95%CI)	P-value	HR (95%CI)	P-value	HR (95%CI)	P-value	HR (95%CI)	P-value
**Gender**	Male vs Female	..	..	0.8 (0.26–2.54)	0.725	1.2 (0.68–2.10)	0.525	1.2 (0.81–1.84)	0.340	..	..	1.9 (0.13–27.75)	0.634	0.4 (0.11–1.09)	0.072
**Age, years**															
	18–34	1Rif	-	1Rif	-	1Rif	-	..	-	1Rif	-	1Rif	-	1Rif	-
	35–49	1.2 (0.13–11.23)	0.874	1.1 (0.48–2.75)	0.746	1.4 (0.70–2.78)	0.337	1Rif		0.4 (0.05–3.69)	0.450	0.3 (0.02–3.41)	0.327	1.6 (0.44–5.56)	0.486
	50-max	..	..	1.50 (0.51–4.46)	0.462	1.4 (0.66–3.09)	0.365	2.1 (1.05–4.21)	0.036	0.2(0.02–1.61)	0.120	..	..	0.4 (0.04–4.18)	0.458
**CARTTherapy**	Yes vs No	..	..	0.3 (0.15–0.78)	0.011	0.6 (0.39–1.06)	0.086	1.5 (0.59–3.66)	0.398	..	..	..	..	0.5 (0.12–1.74)	0.258
**CD4+ count at diagnosis, cell/mm^3^**	0–199 vs 200-max	13.0 (1.36–124.10)	0.026	4.0 (1.73–9.42)	0.001	2.2 (1.31–3.10)	0.001	2.0 (0.99–3.96)	0.050	..	..	0.8 (0.06–8.95)	0.821	2.6 (1.01–6.44)	0.046
**IVDU**	Yes vs No	..	..	3.2 (1.53–6.55)	0.002	..	..		..	1.6 (1.13–2.33)	0.008	2.1 (0.18–24.64)	0.559	..	..
**HIV RNA at diagnosis**	Positive vs Negative	..	..	..	..	1.7 (0.96–3.00)	0.071		..		..	..	..	..	..

Note: ADCs: AIDS-defining cancers, NADCs: Non-AIDS-defining cancers, IVDU: injection drug users, cART: combined antiretroviral therapy, CA: cancer, KS: Kaposi Sarcoma; NHL: non Hodgkin lymphoma; HL: Hodgkin lymphoma; HR: Hazard ratio; CI: confidence interval.

HRs and 95%CIs from the most parsimonious Cox models for ADC and NADC specific cancers. We used the stepwise backward procedure to eliminate non-significant variables. The “..” denotes the variables that were eliminated from the models in the process of stepwise backwards elimination, and factors that were not evaluable for some cancers.

## Discussion

Our results, from a large, multicenter Italian HIV-infected cohort, describe survival after cancer diagnoses in patients from 1998–2012. Cancer occurrence has increasingly contributed to overall mortality among HIV-infected populations [20]–[22]. We found that around 6% of HIV-infected patients developed malignancies (900 malignancies) and almost 40% of these patients were dead at the time of analysis.

Although no differences in survival were observed between ADC and NADC categories during the first 2 years after cancer diagnosis, with a mortality rate close to 30% for both of them, the overall survival after a NADC diagnosis was poorer than after an ADC diagnosis and varied substantially depending on the type of NADC. Moreover, only 45% of patients with NADC were alive 10 years after cancer diagnosis, compared with 60% of those with ADC. Notably, patients with NADCs had a better immunological status than patients with ADCs at the time of cancer diagnosis with statistically significant higher nadir and CD4+ T-cell counts, indicating that patients with NADC appeared to have inferior survival despite better immunity.

Considering the year of diagnosis, we observed that earlier period of NADC diagnosis was associated with a poorer prognosis. This probably reflects the extended use of cancer therapy in HIV-infected people in recent years or improved screening of these patients leading to a diagnosis of early-stage disease (data about cancer screening and cancer staging are not available in the Master cohort). Similar association was reported in other studies [22]–[23]. On contrary, we found no differences in survival after ADCs depending on period of diagnosis (p = 0.218). Since the introduction of effective HIV treatment, there has been an improvement in the control of HIV replication and greater CD4+ T-cell count increases. Therefore patients with ADCs have a better prognosis, although a more advanced immunodeficiency status is still the dominant risk factor for death [24]. Recently, a study within the Collaboration of Observational HIV Epidemiological Research Europe (COHERE) cohort described that persons with HIV infection are not fully immune reconstituted until CD4 counts increased to >750 cells/mm3. Therefore they remain at AIDS events risk, although values >500 cells/mm3 give them a good immunity [25]. This improvement in viro-immunological markers and the younger age of patients at ADC diagnosis could in part explain the higher survival rates we observed in patients after an ADC diagnosis. As previously described, after a cancer diagnosis the mortality increases with age [20]. A better control of HIV-related factors is therefore a key factor to continue improving survival of patients who develop ADCs even in the era of cART.

Moreover, our results show variations in survival among types of cancer within the two categories, NADCs and ADCs. In particular, we found that NHL for ADC and lung cancer for NADC had the poorest prognosis. Consistent with other studies, we observed a five-year survival of approximately 50% for NHL [15], [26] and a one-year survival of just 30% for lung cancer [27]. The shift toward less biologically favorable and curable lymphomas could in part explain the poor survival after NHL even in the cART era. Indeed, a reversal in incidence of different lymphomas during the last years have been reported: incidence of Burkitt's lymphoma increased, whereas diffuse large B-cell lymphoma and primary central nervous system lymphoma decreased [28]. For lung cancer, as in HIV-negative cases, the clinical stage of cancer is highly predictive of survival, and long-term overall survival can only be achieved at the limited stages [27].

Liver cancer is mainly driven by hepatitis coinfection in the HIV-infected patient as a late complication of liver cirrhosis [30]. Indeed, all patients diagnosed with liver cancer in our study were coinfected with hepatitis virus. Potent cART has improved the survival of HIV-infected individuals long enough to allow liver cancer to emerge in patients with known risk factors for liver cancer, such as prolonged ethanol consumption or chronic viral hepatitis [30]. Moreover, the management of liver cirrhosis by clinicians attending HIV-infected patients has probably improved in the last decade, resulting in longer survival of HIV-infected patients with cirrhosis. Compared to the data of the Italian cancer registries (the Italian Association of Cancer Registries AIRTUM) [29], we observed an increased 5 years survival for HIV patients with liver cancer (relative survival rate: 15% *vs* 32%, respectively). Improvements in liver cancer prognosis could be due to a combination of diagnostic anticipation and better control of disease progression. On contrary, we observed a poorer survival for HIV-infected patients with HL (5-years relative survival rate: 58%) respect to the general population with HL (5-years relative survival rate: 83%). This result could be in part explained by the differences in clinicopathological characteristics of HIV-related HL (HIV-HL from those of HL in HIV-uninfected population [31]. In fact, HIV-HL is characterized by a more aggressive clinical presentation, with an unfavorable histological subtype as opposed to the subtype observed in HIV-negative young adults. The five-year relative survival (age and gender-standardized) in the Italian general population for the cancers diagnosed between 2000 and 2004, and the 5-years relative survival in the master cohort for the cancers diagnosed in the period 1998–2012 are reported in the Table S1.

HIV-related immunosuppression is a well-accepted, strong biological risk factor for the virus-associated cancers of AIDS-defining malignancies. As expected, among ADCs, viro-immunological variables and HIV treatment at cancer diagnosis influenced the prognosis of HIV-positive patients diagnosed with Kaposi sarcoma and NHL. Many important cancer sites have benefited from screening [32]–[33]. In our study, cervical cancer and breast cancer, both malignancies in which screening programs have been introduced in general population and more specifically during the follow-up for HIV infection, are the cancers with the better prognosis. Survival rates after cervical cancer were statistically significantly lower in patients with CD4+ T-cell count less than 200 cell/mm^3^ at cancer diagnosis, although in our cohort the proportion of patients with CD4 counts above 200 cell/mm3 at cervical cancer diagnosis was higher compared with patients with other AIDS-related malignancies (p = 0.005). Different studies showed that women with AIDS related cervical cancer differ from women with other HIV related malignancies in two ways: they had less immune suppression and the cause of death was more likely to be attributed to cancer than to opportunistic infections [34]–[35]. Moreover, data in the literature have shown that the clinical course of cervical cancer becomes more aggressive when the CD4+ T-cell count is low [36]. Differently from the other ADCs, in our study cervical cancer survival seemed to be unaffected by the use of cART.

Importantly, low CD4+ T-cell count at NADC diagnosis was strongly associated with poor survival for all NADC combined and in particular for Hodgkin lymphoma, emphasizing the need for timely HIV treatment. For liver cancer, the association between the CD4 count at the time of cancer diagnosis and mortality risk was next to threshold for statistical significance (p = 0.05). For breast and lung cancers we found no association between HIV clinical variables (cART, CD4 count, HIVRNA) and subsequent mortality, indicating that HIV may have no effect on these malignancies.

The cumulative survival probabilities after a malignancy- both ADCs and NADCs- were worse in patients with a history of IVDU. Notably, a history of IVDU was an important risk factor for survival after lung cancer diagnosis. Indeed, HIV transmission mode is an important predictor of prognosis in HIV-infected persons and different studies [25], [37]–[38] showed that IDVU is strongly associated to AIDS events even with CD4 counts >500 mm3. IVDU may be also a marker for other lifestyle habits (e.g. smoking habits, alcohol intake) that can influence the risk of death. Moreover, adherence to antiretroviral therapy among IVDU is often suboptimal. Finally, we found that this factor had a more negative impact on survival rates of HIV-infected patients respect to the absence of cART at cancer diagnosis. Interestingly, in our study cART at the time of cancer diagnosis seemed to influence ADC but not NADC prognosis.

Our study has several limitations. First, the data were retrospectively collected and so it is possible that the number of cancer diagnoses and death were underestimated. Second, information regarding the life-style of patients (tobacco exposure, alcohol abuse), cancer stage and treatment, and causes of death were not available in this study. However, it is important to underline that the assessment of cancer as cause of death among subjects with HIV/AIDS is complicated, as they frequently show several concomitant serious medical condition (immunodeficiency secondary to chemotherapy, interruption of cART due to chemotherapy interaction or increased of secondary effects, etc.). The survival analysis was not performed for any type of NHL although they have different prognosis. However, our data are consistent with those reported in other cohort study that evaluate survival for all NHL combined [15] and for diffuse large-cell lymphoma [26]. The small number of cases for some cancers also is a limitation; in particular, the study included around 30 patients with cervical, breast or lung cancer. Finally, just a few patients reached a follow-up ≥10 years (median follow-up was 4.58 years for patients with an ADC diagnosis and 3.35 years for those with a NADC diagnosis) and it was too small/short to determine the related mid- or long term survival probabilities. Despite these limitations, a strength of our work is the long-term follow-up, including both person/years and median years of follow-up.

Taken together, findings from this large prospective study of survival in HIV-infected patients suggest that maintaining higher CD4+ T-cell counts is a key factor to improve prognosis after both ADCs and NADCs, in particular when considering Kaposi sarcoma and the lymphomas. On contrary CD4 The survival after diagnosis of NADCs is poorer than after ADCs but has shown an improvement in the last years. Importantly, patients with a history of injection drug use represent the population with the worst survival after a cancer diagnosis and therefore these patients should be targeted with screening and preventative strategies. Survival studies on cancer in HIV-infected patients can help to describe an important phenomenon, giving an indication on overall access to early diagnosis and diffusion of screening interventions, and no less importantly, quality, equity and response to cancer treatments with respect to HIV-uninfected patients.

## Supporting Information

Table S1 **The five-year relative survival (age and gender-standardized) in the Italian general population for the cancers diagnosed between 2000 and 2004, and the 5-years relative survival in the Master cohort for the cancers diagnosed in the period 1998–2012.**
(DOCX)
