# Supplementary material for: Survival in HIV-Infected Patients after a Cancer Diagnosis in the cART Era: Results of an Italian Multicenter Study
Source: PLoS One. 2014 Apr 23;9(4):e94768. doi: 10.1371/journal.pone.0094768 (PMC3997420; doi:10.1371/journal.pone.0094768)
Supplement: Table S1 — The five-year relative survival (age and gender-standardized) in the Italian general population for the cancers diagnosed between 2000 and 2004, and the 5-years relative survival in the Master cohort for the cancers diagnosed in the period 1998–2012. (DOCX) [file pone.0094768.s001.docx]

**Table S1: 5-year relative survival**

| Observational-Time | 1998-2012 | 2000-2004 |
| --- | --- | --- |
| Cancer Type or site (ICD-10) | **5-years survival in the Master Cohort (standardized for age and gender)** | **5-years survival in the Italian general population (AIRTUM) (standardized for age and gender)** |
| AIDS-defining cancer | | |
| Kaposi sarcoma | 80 (73-85) | 87 (83-90) |
| Non-Hodgkin lymphoma | 55 (48-62) | 60 (59-60) |
| Cervical cancer | 88 (70-95) | 68 (66-69) |
| Non AIDS-defining cancer | | |
| Liver cancer | **32(20-45)** | **15 (15-16)** |
| Hodgkin lymphoma | **58(42-71)** | **83 (81-84)** |
| Lung cancer | 30 (15-47) | 14(14-14) |
| Breast cancer | 79 (55-91) | 85(85-85) |

**Note: the five-year relative survival (age and gender-standardized) in the Italian general population for the cancers diagnosed between 2000 and 2004, and the 5-years relative survival in the Master cohort for the cancers diagnosed in the period 1998-2012.**
